# All-Fiber Measurement of Surface Tension Using a Two-Hole Fiber

**DOI:** 10.3390/s20154219

**Published:** 2020-07-29

**Authors:** Jose R. Guzman-Sepulveda, Daniel A. May-Arrioja, Miguel A. Fuentes-Fuentes, Natanael Cuando-Espitia, Miguel Torres-Cisneros, Karina Gonzalez-Gutierrez, Patrick LiKamWa

**Affiliations:** 1 Center for Research and Advanced Studies of the National Polytechnic Institute (CINVESTAV Unidad Monterrey), Apodaca, Nuevo Leon 66600, Mexico; jose.guzmans@cinvestav.mx; 2 Fiber and Integrated Optics Laboratory, Centro de Investigaciones en Óptica A.C., Aguascalientes, AGS 20200, Mexico; fuentesma@cio.mx; 3 CONACyT, Applied Physics Group, DICIS, University of Guanajuato, Salamanca, GTO 368850, Mexico; natanael.cuando@gmail.com; 4 Applied Physics Group, DICIS, University of Guanajuato, Salamanca, GTO 368850, Mexico; torres.cisneros@ugto.mx; 5 Motorola Solutions de México, Cuajimalpa, CDMX 05120, Mexico; karinag810@gmail.com; 6 CREOL, The College of Optics and Photonics, University of Central Florida, Orlando, FL 32816, USA; patrick@creol.ucf.edu

**Keywords:** surface tension, specialty fiber, fiber optic sensor, optofluidic sensor, remote sensing, optical sensing and sensors

## Abstract

An all-fiber approach is presented to measure surface tension. The experimental realization relies on the use of a specialty fiber, a so-called two-hole fiber (THF), which serves a two-fold purpose: providing a capillary channel to produce bubbles while having the means to measure the power reflected at the end facet of the fiber core. We demonstrate that provided a controlled injection of gas into the hollow channels of the THF, surface tension measurements are possible by simply tracking the Fresnel reflection at the distal end of the THF. Our results show that the characteristic times involved in the bubble formation process, from where the surface tension of the liquids under test is retrieved, can be measured from the train of pulses generated by the continuous formation and detachment of bubbles.

## 1. Introduction

Traditionally, interfacial tension is measured through the measurement of forces, pressure differences, or deformations [1,2,3]. Common methodologies consist of partially immersing tubes, plates, or rings into the liquid of interest and then retrieving the surface tension from the characteristics of the meniscus at equilibrium, e.g., from the angle of the contact line at the point of maximum wetting height. In situations where the measurement is required to be performed on smaller volumes, deformation-based measurements can be advantageous, since surface tension can be inferred from the profile of microliter-sized droplets that can be pending, sessile, or rotating. Compared to the previous approaches, these perform on a small sample volume at the expense of requiring a more accurate experimental control due to the significant sensitivity to the conditions in which the measurement is performed, e.g., surface hydrophobicity.

More recently, surface tension measurements based in microfluidic devices have been reported using straight channels [4,5] as well as junctions [6,7], where the formation of bubbles/droplets is induced by the controlled injection of gas. In a way, this methodology is a variant of the maximum bubble pressure (MBP) method, which is performed at smaller spatial scales. In microfluidic-based approaches, the surface tension characteristics of the liquid inside the micro-channel can be inferred from either the morphology of the droplet or the rate of bubble formation. In this regard, it has been demonstrated that a simple bubble counting method can be achieved by instrumenting the micro-channel with optical fibers that allow evaluating the transmission of light across the channel [4]. Some recent improvements in this area include the capability for ultralow surface tension measurement by tracking the transit trajectory of magnetic particles across the interface of two liquids inside the micro-channels [8] and the in situ manipulation of surfactant gradients along the micro-channel in order to test the different interfacial tension conditions in a single measurement [9]. In these approaches, imaging systems with large spatial resolution are required either to track the bubbles accurately or to follow the evolution of the bubble profile as they travel along the channel. Other versions of microfluidic devices are those based on engineered individual micro-pores [10] and pore arrays [11,12]. Analogously to the previous case, the pore-based platforms resemble the capillary rise method at smaller scales.

In the approaches mentioned above, optics assists the measurement mainly by providing imaging capabilities. For instance, in the case of a droplet profile measurement, an imaging system is required to measure the shape of the droplet’s shadow that is cast onto a camera. Even in the case of microfluidic architectures, optical fiber-based instrumentations serve the purpose of counting the bubbles by merely assessing the transmission of light across the micro-channel, which can also be performed by using an imaging system that allows seeing the bubbles from the top.

Recently, a paradigm change occurred when optical structures, whose response depends non-trivially on the surface tension, were used to measure the surface tension of liquids. For example, the transmissivity/reflectivity of in-fiber Fabry–Perot (FP) cavities have been associated with the interfacial surface properties of liquids involved in the architecture [13,14]. In the first of these approaches [13], the FP cavity is formed by a remnant pendant drop that attaches to the end facet of an optical fiber after the fiber was previously immersed into the liquid and then extracted from it. In this case, the shape of the pendant droplet, and thus the thickness of the FP, depends on the particularities of the wetting process for the specific liquid used. This approach significantly relaxes the sample volume requirements, since all that is needed is a small droplet to be deposited at the tip of the fiber. In a way, this approach is similar to the micro-scale version of the pendant droplet method, with the difference that in this case, the parameter of interest is the effective thickness of the droplet (as opposed to the time evolution of the contour line). In the second approach [14], an FP cavity is formed at the tip of an optical fiber that is attached to a fiber-sized capillary tube (hollow optical fiber). Similarly to the previous case, the length of the cavity, and thus its light transmission/reflection characteristics, depends on the surface tension of the liquid under investigation. However, as opposed to the previous case, here, the medium inside the FP cavity is air. In a way, this is a micro-scale version of the capillary rise method.

Here, surface tension measurements are demonstrated based on an all-fiber configuration by simply tracking the time-dependent reflection at the tip of a two-hole fiber (THF), which is immersed in the liquid under study. The cross-sectional structure of the THF can be described as a standard single-mode fiber (SMF) with two holes disposed to the sides of the core. Gas is injected controllably via the hollow channels of the THF in order to induce the continuous formation and detachment of bubbles at the distal end of the THF. At constant injection pressure, the periodic rate of bubble formation is constant and related to the liquid surface tension where the THF is immersed. Our experimental implementation is basically a time-resolved refractometer working in reflection, where (1) the same optical fiber is used to inject and collect light, and (2) lower and higher reflection correspond to the THF–liquid interface and the THF–gas interface, respectively. In this way, we achieve a self-contained configuration where the THF is used for both bubble formation and time-resolving the Fresnel reflections at the THF tip, without needing actually to image the bubbles. Our scheme can be thought of as a micro-scale version of the traditional bubble-based methodology. Our results show that the characteristic times that are directly related to the formation of bubbles, and therefore the surface tension of the liquids under test, can be inferred from the train of pulses generated by the continuous creation and detachment of bubbles. We should also highlight that our all-fiber implementation preserves the inherent benefits of the use of optical fibers such as immunity to electromagnetic interference, multiplexing capabilities, and the ability to operate in harsh environments.

## 2. Principle of Operation: An Analogy with the Maximum Bubble Pressure Method

**Figure 1:**
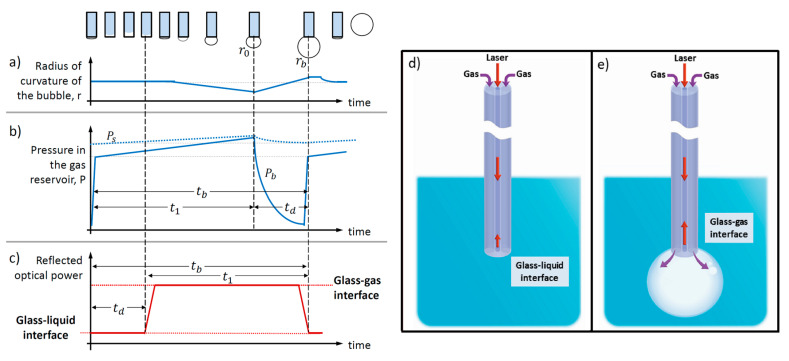
Schematic diagram of the concept: in a typical maximum bubble pressure (MBP) measurement, the characteristic times involved in the process of bubble formation, from where the surface tension is inferred, can be measured by following the time evolution of either (**a**) the radius of the bubble or (**b**) the gas reservoir pressure. (**c**–**e**) The same characteristic times can be retrieved by following the time evolution of the optical power reflected from a small, partially transmitting interface located at the center of the capillary that is used to induce the formation of the bubbles. In that case, the continuous creation and detachment of bubbles due to the injection of gas at a constant rate will produce a train of pulses in the reflected signal (**c**) due to the difference in the reflectivity between (**d**) the glass–liquid interface versus (**e**) the glass–gas interface, which is around one order of magnitude for aqueous liquids. Similarly to an MBP measurement, the surface tension of the liquid under test can be measured indirectly from the characteristic times retrieved, i.e., from the characteristic periodicity of the train of pulses generated. Panels (**a**,**b**) were adapted from Refs. [15,16,17] (see text for details).

Figure 1 shows the general process of bubble formation schematically, and some associated parameters used to describe it in an MBP experiment. The bubble is implicitly assumed to be formed inside a liquid medium, which is semi-infinite and homogeneous, and it has a surface tension γ. Specifically, Figure 1a,b illustrates the temporal evolution of the bubble radius, r, as well as the pressure both inside the bubble, $P_{b}$, and in the measuring system, $P_{s}$, respectively (schematic adapted from Refs. [15,16,17]). These are the parameters typically followed in an MBP measurement [17,18].

Traditionally, the bubble growth is divided into two intervals: the lifetime, $t_{1}$, which includes the time from when the bubble starts to form until the maximum bubble pressure is achieved (at this point the radius of the bubble is $r=r_{0}$); and the dead time, $t_{d}$, which relates to the subsequent bubble growth until the bubble detaches ($r=r_{b}$) [19,20]. In this context, the surface tension is directly related to the lifetime, and, ideally, one would prefer to measure it directly; however, this is not an easy task mainly because of the small pressure changes induced in the gas reservoir, which requires high-sensitivity pressure sensing. In fact, in an MBP measurement, one calculates the lifetime indirectly from the difference between the bubble time (total duration between subsequent bubbles, $t_{b}=t_{1}+t_{d}$) and the dead time, $t_{d}$, i.e., the lifetime is obtained from $t_{1}=t_{b}-t_{d}$. Both $t_{b}$ and $t_{d}$, in turn, are measured via the time evolution of the pressure in the gas reservoir, while assuming a laminar gas flow within the capillary (Poiseuille regime) and a spherical bubble growth [16,21]. Therefore, an accurate determination of $t_{d}\;$ is critical. This becomes more evident in dynamic measurements where the surface tension is time-evolving, e.g., the bubble’s surface is aging [15,17,22].

Here, we propose a new approach to measure the characteristic times associated to the bubble formation process: by following the time evolution of the optical power reflected from a small, partially transmitting interface located at the center of the capillary that is used to induce the formation of bubbles (Figure 1c). In our geometry, that interface arises naturally at the end facet of the core of the THF, and it is located at the center of the ‘effective capillary’ formed by the two hollow channels of the THF (Figure 1d,e). The area of the small interface is determined by the core size of the THF, which is approximately $50{\;\mu m}^{2}$. Figure 1c shows schematically the time evolution of the optical power reflected at the end facet of the fiber, due to inherent Fresnel reflections, which correspond to the case of glass–liquid (Figure 1d) and glass–gas interfaces (Figure 1e), as indicated. For instance, the refractive index (RI) of the THF is close to 1.45, the RI of the gas can be taken as $n_{g}=1.0$, and the extreme values of the RI of the samples are $n_{w}=1.333$ (water) and $n_{IPA}=1.358$ (isopropanol 35%) at a wavelength of 1550 nm. Therefore, if we calculate the Fresnel reflectivity at normal incidence between the THF and the scenarios when we have and we do not have a bubble, we can verify that the ratio between the reflectivity of such scenarios is around one order of magnitude, which is enough to observe the power changes in a standard photodiode and oscilloscope. If we correlate the duration of the time intervals in Figure 1c with those in Figure 1b, we can notice that we should be able to retrieve the lifetime, dead time, and bubble time by following the time evolution of the reflected power. Overall, our approach allows measuring directly the characteristic times involved in the process of bubble formation, without having to monitor the small pressure changes produced in a gas reservoir. From an experimental standpoint, this relaxes the need for a large sensitivity to small pressure differences while still providing means to determine those times accurately.

## 3. Materials and Methods

### 3.1. Experimental Setup

**Figure 2:**
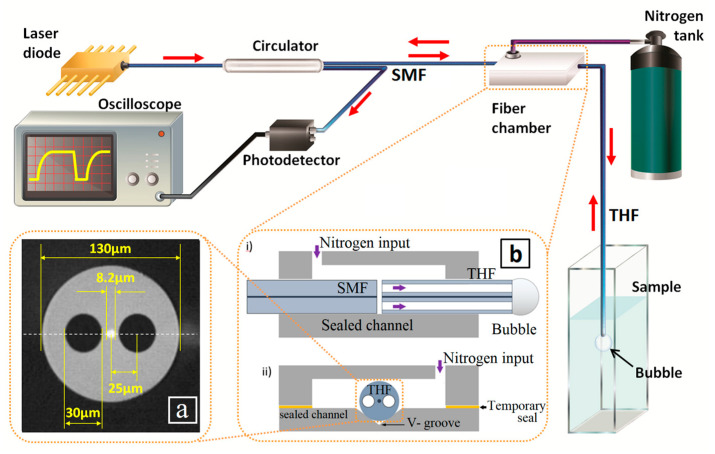
Schematic of the experimental setup used for the all-fiber measurements of surface tension, which consists of a time-resolved, fiber-based refractometer working in reflection. Light from a fiber pigtailed laser diode is coupled to the two-hole fiber (THF) using standard single-mode fiber (SMFs); the reflected signal is directed to the photodetector through the circulator. (**a**) Microscope photograph of the cross-section of the THF used in this work; the fiber was fabricated at ACREO Fiberlab. (**b**) Schematics of the lateral view and front view of the pressure chamber that allows gas to flow through the holes of the THF, in order to form bubbles at its distal end, at a controlled rate. Inside this chamber, the SMF and the THF are efficiently butt-coupled using a v-groove to align the two fibers.

Our experimental setup is shown in Figure 2. As previously mentioned, essentially, our implementation is a time-resolved, fiber-based refractometer working in reflection, which is constructed using SMFs and a section of THF at the distal end of the fiber arrangement. The core of the THF is located at the center of the fiber, as shown in Figure 2a, and its diameter is similar to that of a standard SMF. Therefore, an efficient THF–SMF joint can be achieved by simple butt-coupling, e.g., by aligning the fibers inside a v-groove, as schematically shown in Figure 2b. The arrangement shown in Figure 2b allows not only for sending and collecting light through the SMF–THF arrangement but also for injecting gas into the THF for a controlled bubble formation. After a small separation between the THF and SMF is achieved by mildly butt-coupling them, both fibers are fixed in order to maintain the same pressure as well as the magnitude of the reflected signal through all the experiments. Nitrogen was the gas used in our experiments, and the applied pressure was controlled and measured using a needle valve and manometer, respectively. The THF was manufactured at ACREO Fiberlab (Kista, Sweden).

In our implementation, a Fresnel reflection corresponding to a glass–gas interface is detected when the bubble is present at the tip of the THF; correspondingly, a glass–liquid interface is seen prior to the bubble formation. Therefore, a train of bubbles with a specific periodicity is generated by maintaining a constant pressure, which is dictated by the liquid surface tension being measured by the THF (see Figure 1c). In this way, a micro-scale version of the bubble pressure method can be achieved, where gas bubbles are generated in the liquid under study, at a constant rate, through a partially immersed capillary tube of known inner diameter. Additionally, our scheme does not require movable parts; also, it relaxes the requirements of the sample volume due to the small size of the bubbles that are induced, and it allows using the same optical fiber for injecting the gas and performing the optical measurement.

Light from a fiber pigtailed laser diode (SFL1550S, Thorlabs, Newton, MA, USA), operating at a wavelength of 1550 nm and 1 mW optical power, is coupled to the SMF–THF arrangement through a circulator (DKPhotonics, Shenzhen, China), as schematically indicated in Figure 2. The light reflected from the distal end-facet of the THF is efficiently collected and measured with the photodetector (PDA10CS, Thorlabs, Newton, MA, USA). An oscilloscope (TDS1001B, Tektronix, Beaverton, OR, USA) is used to record the signal measured with the photodiode. Our measurement scheme includes a lock-in detection system (SR510 Lock-In Amplifier, Stanford Research Systems, Sunnyvale, CA, USA), which allows not only improving the quality of the signals measured, but also suppressing the thermal effects due to light absorption, even for an infrared operating wavelength. More specifically, we modulated the input laser at a frequency of 1 kHz (full modulation depth at 50% duty cycle) and set the lock-in detection system accordingly. With these settings, the temperature increase induced due to absorption is negligible for all samples (see Appendix A).

### 3.2. Fabrication of the Device

The fabrication of our sensing architecture involves the use of standard commercial-grade SMFs (SMF-28) for coupling the light to and from the measurement system. Initially, the THF has a diameter of 130 µm (see Figure 2b). Thus, in order to align the cores of the SMF and the THF in the v-groove, we first removed some material from the cladding of the THF. In order to do so, we immersed the THF in a commercial solution of hydrofluoric acid with surfactant (buffered oxide etching, BOE, 6:1; etching rate of 130 nm/min [23]) during 19 min, for a final diameter of 125 μm. After etching, both fibers were mounted on translation stages and then placed on a homemade platform with a v-groove. Finally, their alignment was optimized by maximizing the reflected power with the aid of micro-position controls of the translation stages.

Once the fibers were properly butt-coupled and the reflection from the assembly was optimized, the pressure chamber was sealed, in order to avoid gas leaks and ensure that the input pressure is known.

### 3.3. Sample Preparation

In our proof-of-concept experiments, we used binary mixtures of water and isopropyl alcohol (2-propanol; IPA). These aqueous solutions are standard liquids that can be used for calibration, since their surface tension properties are well-known. At a constant temperature, the surface tension of these solutions decreases with increasing alcohol concentration nonlinearly: the change in surface tension caused by a given change in alcohol concentration is more significant at lower concentrations [24]. We prepared solutions with IPA concentration from 0 wt % to 35 wt %, which allows us to reduce the maximum surface tension value in almost equal steps, as shown in Table 1. The maximum concentration of 35 wt % was chosen in order to test a maximum reduction of approximately 60% in the value of the surface tension, with respect to the case of pure water (27.95 mN-m^−1^ for 35 wt % IPA versus 70.91 mN-m^−1^ for pure water) [24,25,26]. This range is sufficient to cover the portion of the concentration-dependent surface tension curve, where the more significant changes occur [24].

## 4. Results

### 4.1. Observation of the Bubbles

**Figure 3:**
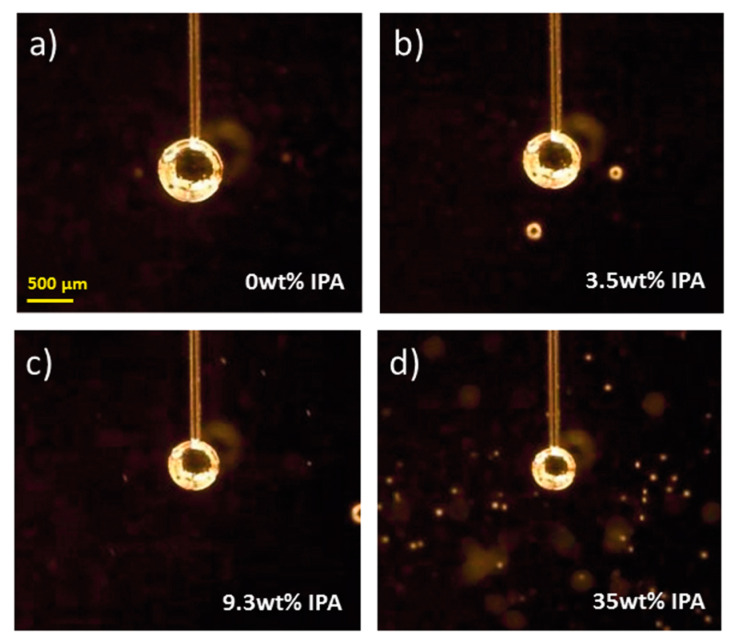
Digital photographs of the bubbles, at the time when the maximum size is reached, for different concentrations of isopropyl alcohol. As indicated the content of IPA is (**a**) 0 wt %, (**b**) 3.5 wt %, (**c**) 9.3 wt %, and (**d**) 35 wt %. The scale bar is 500 μm.

Similarly to microfluidics measurements, where the frequency of bubble/droplet formation depends on surface tension (it decreases with increasing surface tension at constant pressure), in our case, the bubble lifetime also depends on the surface tension in a non-trivial manner. A larger surface tension leads to a longer bubble lifetime, and thus to a lower frequency of bubble formation, since the bubble diameter grows with increasing surface tension, i.e., liquids with larger surface tension can grow bigger bubbles, which takes more time, at constant pressure, than for a liquid with lower surface tension. Figure 3 shows pictures of the maximum bubble diameter formed for the different concentrations of IPA, as indicated.

### 4.2. Determination of the Dead Time

First, we verified the validity of the two main approximations on which this work relies: (1) the spherical bubble growth, and (2) the inertia-less regime. We found that both approximations are amply satisfied in all the experimental conditions studied here (see Appendix B).

Within the frame of the Poiseuille approximation, the growth of the bubble’s radius, r, as a result of the pressure difference between the two ends of a capillary can be expressed by a differential equation as [15,16,17,21] $\frac{dr}{dt}=\frac{r_{0}^{4}\left(\Delta P-2\gamma /r\right)}{32\;l\;\eta \;r^{2}}$, where l is the length of the capillary, η is the dynamic viscosity of the gas, $r_{0}$ is the radius of the capillary, and $\Delta P=P_{s}-P_{h}$ is the measured maximum pressure in the bubble; $P_{s}$ is the pressure of gas injection and $P_{h}=\Delta \rho gh$ is the hydrostatic pressure, with $\Delta \rho =\rho_{liquid}-\rho_{gas}$ being the density difference between the gas and the liquid, g is the gravity, and h is the depth within the liquid at which a bubble is formed.

One can obtain an analytical expression for the dead time by integrating this equation over the time that it takes for the bubble to increase from $r_{0}$ to the radius $r_{b}$ at the moment of detaching [15,16,17,21], $t_{d}=\frac{32\;l\;\eta }{r_{0}\Delta P}\left[\frac{1}{3}{\left(\frac{r_{b}}{r_{0}}\right)}^{3}+\frac{\gamma^{*}}{r_{0}\Delta P}{\left(\frac{r_{b}}{r_{0}}\right)}^{2}\right]$ (see Figure 1). In this equation, the gas expansion into the infinite space is described by the first term on the right-hand side, and the second term corresponds to the capillary pressure in the growing bubble. In the last equation, a growing bubble will experience a surface tension given by $\gamma^{*}$ during the dead time. In most practical cases, the variation of $\gamma^{*}$ has a small impact on $t_{d}$; thus, $\gamma^{*}$ can be replaced by γ [21].

### 4.3. Estimations of the Dead Time in our Results

In the experiments, we used standard rectangular cuvettes (10 mm × 10 mm × 45 mm) for the different samples. The tip of the THF was introduced into the liquid and placed at a depth of 10 mm, at which the hydrostatic pressure is $P_{h}\approx 100\;Pa$. Even though this pressure does not seem to be significant, it is comparable to the injection pressure required for the bubbles to be induced slowly (with characteristic times in the range of tens of ms to seconds) in such small capillaries, which is actually desired in order to safely neglect inertial effects.

Nitrogen was used as the gas through all the experiments ($\eta =17.58\times 10^{-6}\mathrm{Pa}-s)$, and the minimum detectable pressure of $P_{s}\approx 0.05\;psi=340\;\mathrm{Pa}$ was used in our experimental setup (ΔP≈240 Pa). The length of the capillary is assumed as l=1 m, and its size is correlated with that of the THF, i.e., $r_{0}\approx r_{THF}=62.5\;\mu m$.

**Figure 4:**
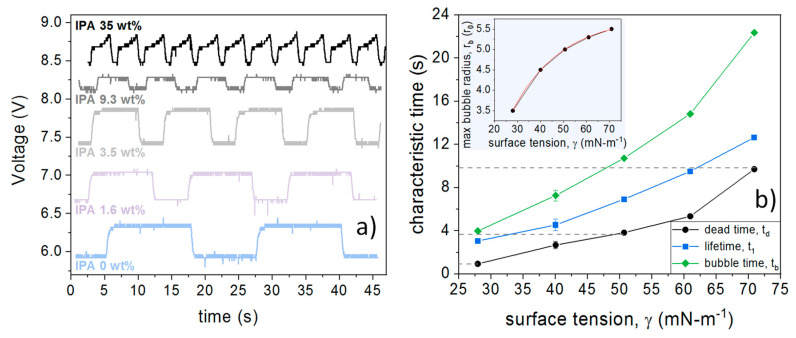
(**a**) Voltage measured by the photodetector for different binary mixtures as a function of time. (**b**) Characteristic times obtained from the data in panel (**a**); with all the existent periods averaged during total measurement time of 45 s; the error bars indicate the standard deviation. Inset: maximum bubble radius, in units of the capillary radius, versus surface tension estimated from the photographs in Figure 3; this plot is the ratio $r_{b}/r_{0}$ that is necessary to retrieve the value of the surface tension from the rigorous theory (see the calculations of the dead time above for details). The dashed lines in panel (**b**) indicate the values of the dead time retrieved from the rigorous analysis, which are in good agreement with those measured in our experiments.

The voltage measured by the photodetector for different binary mixtures, and over a certain period of time, is shown in Figure 4a. For these measurements, the pressure was set at about 0.05 psi (340 Pa). All of the curves shown in Figure 4a have the same baseline voltage as the IPA concentration of 0%, and they were vertically shifted for better visualization purposes.

While the bubble is present, the voltage measured is larger, as expected from the Fresnel reflectivity at a glass–air interface, as opposed to a glass–liquid one. Again, in this time-dependent voltage trace, $t_{1}$ is considered the time that the voltage remains at a higher value; $t_{d}$ is the time that the voltage remains at a lower value; and $t_{b}$ the time between two subsequent bubbles. We can also notice that the reflectivity of IPA aqueous solutions is slightly reduced when we increase the IPA concentration [26].

From Figure 4a, it can be seen that all the characteristic times increase with increasing surface tension, which directly reflects the higher resistance of the fluid to be deformed by the gas bubble, thus leading to a longer growth duration. It can also be seen that our measurement takes less than a minute for each sample to be tested, even for the largest surface tension explored. Of course, the duration required for the measurement will increase with the increasing surface tension.

The bubble formation process can be better visualized by plotting the characteristic times versus the surface tension of the samples, as shown in Figure 4b. In this figure, the characteristic times are obtained by averaging all of the existent periods during a total measurement of 45 s. Alternatively, we could also plot the characteristic times versus the IPA concentration, which notably resembles the dependence of the surface tension on the concentration [24].

From Figure 4b, the dependence of the surface tension on the dead time can be experimentally retrieved. Basically, the curves in Figure 4b can be fitted to generate a calibration functional form from which the surface tension of unknown samples can be assessed. Nevertheless, in order to complement our experimental results, we now proceed to estimate the dead time for our case by using the rigorous analysis presented above, which requires knowing the maximum size of the bubbles (see Figure 3). The estimation of the maximum bubble radius as a function of the surface tension is shown in the inset of Figure 4b. In this plot, the bubble radius is plotted in units of the capillary radius in order to provide directly the ratio $\left(r_{b}/r_{0}\right)$ that is necessary to retrieve the value of the surface tension from the rigorous theory. The estimations of the bubble radius were done by a simple image analysis of the photographs shown in Figure 3.

In order to illustrate this calculation, we exemplify the retrieval of the dead time for some representative cases. For our baseline condition (deionized water), we observed that $r_{b}\approx 5.5r_{0}$ (see Figure 3a), which leads to a dead time of $t_{d}\approx 9.8\;s$. For the case of an intermediate sample, for instance IPA 3.5 wt %, we observed that $r_{b}\approx 5.0r_{0}$ (see Figure 3b), which leads to a dead time of $t_{d}\approx 3.7\;s$. Finally, for the largest surface tension explored, IPA 35 wt %, we observed that $r_{b}\approx 3.2r_{0}$ (see Figure 3d), which leads to a dead time of $t_{d}\approx 0.9\;s$. These values are actually in good agreement with those measured experimentally, as indicated by the dashed lines in Figure 4b. We should highlight that the estimated values for the dead time, and thus the values for surface tension, are in excellent agreement as compared with the experimental values (Figure 4b).

Finally, in the expression for the estimation of the dead time, the fact that $\gamma^{*}$ can be replaced by γ together with the assumption of the Poiseuille regime leads to a simple relation between the dead time and the bubble time in terms of the gas flow rate, the parameters of the capillary, and the maximum radius achieved before the bubble detachment [15,16,17,21], $t_{d}=t_{b}\frac{L}{K\Delta P}\left(1+\frac{3r_{0}}{2r_{b}}\right)$, where $K=\pi r_{0}^{4}/8\eta l$ is the Poiseuille equation constant (considering a capillary not immersed into the liquid), and L is the gas flow rate. For the above-mentioned parameters of our capillary and the gas used in the experiments, the capillary constant is estimated to be $K\approx 0.34\times 10^{-12}\;m^{3}/\mathrm{Pa}\text{-}s$.

In our case, this expression can be used in different ways. For instance, if the gas flow rate is known, then $r_{b}$ can be estimated without having to image the bubbles, since both $t_{d}$ and $t_{b}$ are measured. However, since in our experiments L is unknown, we can use that expression to estimate the gas flow rate ($t_{d}$, $t_{b}$ and $r_{b}$ are measured), which is around $L\sim 2.5\times 10^{-11}m^{3}/s$ for the cases explored here. It is important to notice that a calculation of L is not trivial due to the geometry of the THF.

## 5. Discussion

Since the invention of the MBP method, the capillary radius has been kept almost constant ($r_{0}\approx 100\;\mu m$) [15]. The reason is that an increase in the capillary radius would decrease the capillary pressure, which in turn would lead to a decrease in the accuracy of the method. The effective size of the capillary formed by the two holes of the optical fiber used here has comparable dimensions to the capillaries typically used in MBP. Thus, in a way, our fiber-based arrangement can be thought of as a micro-scale version of the typical MBP capillary that is instrumented for in situ measurement of the optical reflectivity. This method has proved useful for the evaluation of surfactants effects on surface tension that cannot be resolved in a contact angle measurement for biological fluids where the amount of sample is to be kept small, and also for surfactant or impurities detection, since the measurement remains strongly correlated with concentration for a large range of bubble formation rates [27]. In this regard, our measurement preserves these advantages at the micro-scales where the measurement is performed.

It is also important to note that our approach is suitable to measure the surface tension practically in any liquid, since the measurement relies on the formation of gas bubbles, whose RI is in general much lower than that of the liquids under test. In other words, by using gas bubbles, one can ensure clear discrimination between the reflections from the glass–gas interface versus the gas–liquid interface. In fact, the best contrast in our measurement would be obtained in conditions of RI matching, i.e., in liquids whose RI is close to that of glass, in which the reflection from the glass–liquid interface would be minimal. We should also mention that since the reflected signal has minimal interaction with the liquid being tested, even if the liquid exhibits high absorption at infrared wavelengths, this should not have a detrimental effect on the reflected signal. In fact, it is well known that higher absorption results in a higher Fresnel reflectivity, which will only enhance the reflected signal.

Besides this, the *time-resolved* nature of our measurement makes our approach suitable for performing dynamic measurements of surface tension when the liquid is subjected to non-stationary processes. In that case, the variations of surface tension can be retrieved from the changes in the periodicity of the train of pulses generated. Importantly, in our approach, the frequency of bubble formation can be adjusted by merely tuning the injection pressure, thus allowing us to adequately sample the non-stationary process while having enough pulses for proper averaging.

In future applications, our approach can be modified in several ways. For instance, one can consider flowing liquids through the channels of the THF and measuring the surface tension through the lifetime of microemulsion droplets. In that case, one can anticipate that the reflectivity of the THF will need to be enhanced, e.g., by depositing a thin layer of metal at the end facet of the THF. This would allow not only increasing the reflectivity in general but also performing in conditions of quasi-index-matching while still providing the capability to discriminate between the host liquid and the droplet. Additionally, one can attempt to achieve a more detailed characterization of the bubbles/droplets formed by using a broadband light source and an optical spectrum analyzer instead, in order to perform a time-resolved measurement of the reflection spectrum. This would allow extracting the actual time dependence of the bubble’s size directly, without having to image the bubbles, by modeling the process of bubble/droplet formation as a time-evolving FP cavity.

We should also highlight that the all-fiber configuration presented preserves the advantages of optical fibers such as immunity to electromagnetic interference, multiplexing capabilities, and the ability to operate in harsh environments. This allows sensing remotely in electromagnetically noisy and harsh environments. The fiberized approach also allows performing multi-point measurements, i.e., simultaneous measurements at different locations in a single sample or multiple liquids, by taking advantage of the multiplexing capabilities of optical fibers.

Finally, we would like to mention that from a practical perspective, our measurement is effectively performed in a small volume that is in the order of nanoliters, as determined by the maximum size of the bubbles induced, whose dimensions are comparable to that of the optical fiber used to measure (a few times the size of the cladding of the THF). This makes possible performing measurements that are also *spatially resolved,* with a spatial resolution that is determined by the maximum bubble size that is induced. Having the capability to measure the surface tension at different locations within a sample is relevant in more complex situations where the liquid under study is heterogeneous [28].

## 6. Conclusions

An all-fiber scheme for the measurement of surface tension was proposed and experimentally demonstrated. Our implementation consists essentially of a fiber-based, time-resolved refractometer operating in reflection. A central part of our experimental realization is a THF that allows simultaneously (1) inducing in a controllable manner the formation of bubbles at the THF tip by injecting gas through the holes of the THF and (2) measuring the characteristic times of the processes involved in the formation of bubbles by merely following the reflected power. Importantly, the lifetime, dead time, and bubble time are all measured directly, without needing to image the bubbles. Essentially, our approach allows for a time- and spatially resolved measurement of surface tension.

## Figures and Tables

**Table 1:** Surface Tension of the Binary Isopropyl Alcohol (2-propanol; IPA)–Water Liquid Mixtures Used in this Work.

Concentration of Isopropyl Alcohol (IPA) (wt %)	Weight of Water (g)	Weight of IPA (g)	Surface Tension, γ (mN-m^−1^) @ 25C
**0**	20	0	70.91
**1.64**	19.678	0.32	60.96
**3.5**	19.3	0.7	50.67
**9.3**	18.14	1.86	40.04
**35**	13.0086	6.99	27.95

## Appendix A. Thermal Effects Due to Radiation Absorption

If neither phase transitions nor alterations in the medium take place, then a variation in heat content dQ induces a linear change in temperature dT [29], dQ=mcdT, where m is the mass of the system, and c is its specific heat capacity. In a first-order approximation, heat generation and deposition can be modeled as a two-step process: (1) heat is generated inside the medium during laser exposure; (2) the deposition of heat in the medium is due only to the light that is absorbed. In other words, the absorbed electromagnetic (EM) energy, $E_{abs}$, is completely converted into thermal energy that is transferred to the system i.e., $\Delta Q=E_{abs}$, such that the corresponding change in temperature can be estimated according to [29,30]:

$$\Delta T=\frac{\Delta Q}{mc}=\frac{E_{abs}}{c\rho V_{T}}$$

where the mass has been expressed in terms of the density, ρ, and the total volume of the system, $V_{T}$. The heat deposition per unit volume per unit time (W/m^3^ = J/s/m^3^), due to the heat source inside the medium, is $S=\alpha I_{0}$, where α is the EM absorption coefficient of the medium (in m^−1^), and I is the irradiance of the input beam (in W/m^2^) [29]. Thus, the absorbed energy is $E_{abs}=SV_{H}T$, where $V_{H}$ is the volume where the EM energy is delivered (see the figure below), and T is the duration of the stimulus. In this way, the temperature increase can be estimated as:

$$\Delta T=\frac{SV_{H}T}{c\rho V_{T}}=\left(\frac{V_{H}}{V_{T}}\right)\frac{\alpha I_{0}T}{c\rho }.$$

A summary of the estimations can be found in Table A1 below. The results were obtained using a free-space wavelength of $\lambda_{0}=1.55\;\mu m$; an incident power of $P_{0}=1\;\mathrm{mW}$; and, a radius of the delivering spot (radius of SMF) of $r_{0}=5\;\mu m$. Additionally, we assume that the EM is delivered to a volume $V_{H}$ that has the size of the bubble, and it is much smaller than the size of the sample $V_{T}$. In our experiments, standard quartz cells, with dimensions 10 mm × 10 mm × 45 mm, were filled up completely with the sample such that $V_{T}=4.5\;\mathrm{mL}$. As mentioned in the main text, the measurement involved a lock-in detection system, and the laser was modulated at a frequency of 1 kHz (square signal with full modulation depth; 50% duty cycle). Thus, the duration of the stimulus is of only 0.5 ms; we assume that there is no temperature increase associated to the portion of the cycle where the light source is completely off. As a result, the estimations show that the temperature increase induced due to radiation absorption, per modulation cycle, is negligible; even for the worst case (water), it is less than 0.2 °C.

**Table A1:** Parameters used for thermal effects estimation due to radiation absorption.

	Water	IPA
Density, ρ (kg-m-3)	1000	786
Specific heat capacity, c (J-kg-1-K-1)	4179.6	2680
Imaginary part of the complex RI (refractive index), k @ 1.55 µm	1.475 × 10^−4^	4.389 × 10^−5^
Duration of the stimulus T (s)	5 × 10^−4^	5 × 10^−4^
Bubble radius (r0), where $r_{0}=62.5\;\mu m$	5.5	3.5
EM delivering volume, $V_{H}=\frac{4}{3}\pi r^{3}$ (m^3^)	1.70 × 10^−10^ (170 nL)	0.44 × 10^−10^ (44 nL)
Temperature increase when $V_{T}=4.5\;\mathrm{mL}$$\Delta T_{H}=\left(\frac{V_{H}}{V_{T}}\right)\frac{\alpha I_{0}T}{c\rho }$ (°C)	0.138	0.021

## Appendix B. The Validity of the Approximations of (1) the Spherical Bubble Growth and (2) the Inertia-Less Regime

In a traditional MBP experiment, the surface tension γ is typically estimated using the values of maximum capillary pressure P and the known capillary radius $r_{0}$ via a generalized Laplace equation to [21]:

$$\gamma =\left(\frac{r_{0}P}{2}\right)f$$

where f is a correction factor that takes into account that the bubble will have some deviation from a spherical shape.

In general, the pressure difference between the bubble and the gas reservoir is $\Delta P_{\gamma }=2\gamma /r$, where γ is the surface tension of the liquid and r is the radius of the bubble. At the point of maximum pressure in the bubble, $\Delta P_{\gamma }=\Delta P_{max}=\Delta P_{m}$, thus leading to $\gamma =\left(\frac{r_{0}\Delta P_{m}}{2}\right)f$, where the function f relates to the shape of the bubble’s meniscus.

### Appendix B.1. Spherical Approximation of the Bubble’s Meniscus

This correction factor f can be found in tables [31,32], and it is related to the ratio $r_{0}/a$, where a is the so-called capillary constant defined as a=(2γΔρ g)12. In this equation, γ is the surface tension of the liquid, $\Delta \rho =\rho_{liquid}-\rho_{gas}$ is the difference between the density of the liquid and the gas, and g is the gravity. For all variables given in SI units, a has units of meters. In our case, $\Delta \rho \approx \rho_{liquid}$.

Alternatively, in a more general form, f can be estimated from a power series of the ratio $r_{0}/a$, $f=\underset{i}{\sum }a_{i}{\left(\frac{r_{0}}{a}\right)}^{i}$, with the set of polynomial coefficients $a_{i}$ given by different authors [21].

Regardless of the values of those coefficients, it has been found that (1) for capillaries with a radius $r_{0}<0.2\;\mathrm{mm}$, the value of f differs from unity only insignificantly, and (2) the assumption of sphericity for the bubble is acceptable for small bubbles (<1 mm) when the surface tension predominates other forces [19].

We verified the validity of this assumption from both numerical estimates and experimental observations. Again, a spherical meniscus (f≈1), can be assumed when $\frac{r_{0}}{a}\ll 1$.

#### Appendix B.1.1. Estimation 1

We evaluate the two extreme cases in our experiments, namely the case of pure water and the maximum concentration of IPA, respectively. For the baseline sample (DI water; 0 wt % IPA), $\rho^{\left(0\right)}=1000\;\mathrm{kg}/m^{3}$ and $\gamma^{\left(0\right)}=70.91\times 10^{-3}\;N/m$, while for the sample with the maximum concentration of isopropyl alcohol (35 wt % IPA), $\rho =940\;\mathrm{kg}/m^{3}$ and $\gamma =27.95\times 10^{-3}\;N/m$. This results in values of the capillary constant of $a^{\left(0\right)}\approx 3.80\times 10^{-3}\;m$ and $a\approx 2.46\times 10^{-3}\;m$, respectively. Thus, from this estimation, the assumption of sphericity is satisfied.

#### Appendix B.1.2. Estimation 2

From experimental observations (Figure 3 and inset of Figure 4b), the maximum radius $r_{b}$ is larger than, but comparable to, the diameter of the optical fiber. In a rough estimation, we can take the $r_{0}^{\left(0\right)}\approx 3\;d_{f}=375\;\mu m$ and $r_{0}\approx 1.5\;d_{f}=187\;\mu m$ for the above-mentioned extreme cases, respectively. These results in values of the ratio $\frac{r_{0}}{a}$ in the range between 0.076 and 0.098, which can be considered sufficiently small compared to unity. Thus, from this other estimation, the bubbles can also be assumed to have a spherical meniscus.

### Appendix B.2. Inertia-Less Approximation

The velocity of the gas flow varies due to the pressure decrease inside the growing bubble; thus, in general, one should consider inertial effects [15].

#### Appendix B.2.1. Estimation 1

The condition for neglecting the inertial term in the generalized non-stationary Navier–Stokes equation (see, for instance, Equation (21) in Ref. [15] or Equation (28) in Ref. [17]), and therefore for the applicability of a quasi-stationary regime approximation for the gas flow through the capillary (Poiseuille regime), can be evaluated as the ratio of two characteristic times: the time needed to achieve a uniform pressure distribution in the capillary, $t_{11}≅\frac{8l^{2}}{\pi^{2}N}=\frac{64\eta }{\pi^{2}P_{s}}{\left(\frac{l}{r_{0}}\right)}^{2}$, and the so-called hydrodynamic time of the flowing gas, $t_{h}=\frac{r_{0}^{2}}{8\nu }$ [17]. Specifically, the inertia-less approximation is valid if $\frac{t_{11}}{t_{h}}=8\left(\frac{64\eta }{\pi^{2}P_{s}}\frac{l^{2}}{r_{0}^{4}}\right)\gg 1$, where $P_{s}$ is the pressure at which the gas is injected.

In our case, the kinematic and dynamic viscosity of the gas (nitrogen) is ν=15.27×10−6m2s and $\eta =17.58\times 10^{-6}\mathrm{Pa}\text{-}s$, respectively, and the length of the capillary (length of the piece of fiber) is l≈1−2 m. This leads to $\frac{t_{11}}{t_{h}}≳\frac{1\times 10^{6}\;\mathrm{Pa}}{P_{s}}$, which means that $\frac{t_{11}}{t_{h}}\gg 1$ can be safely assumed for injection pressures smaller than $P_{s}\sim 10^{5\;}\mathrm{Pa}$. In all of our experiments, this condition is amply fulfilled.

#### Appendix B.2.2. Estimation 2

Alternatively, one can evaluate the validity of the inertia-less approximation from the characteristic damping time scale of the sound waves traveling through the gas within the capillary [17,20,33], by evaluating the condition $\omega_{0}\tau <1$, where $\omega_{0}=\left(\pi c_{0}\right)/\left(2l\right)$, $c_{0}$ is the speed of sound in the gas inside capillary, and $\tau =r_{0}^{2}/4\nu$ is the damping time. In terms of the above-mentioned characteristic times, this condition can be expressed as ${\left(\omega_{0}\tau \right)}^{2}=8{\left(t_{11}/t_{h}\right)}^{-1}$ [17]. In our case, we estimated ω0≈5.4×102 rads and $\tau \approx 1.47\times 10^{-5}\;s$. From these estimations, we obtain $\omega_{0}\tau \approx 8\times 10^{-3}\ll 1$ and, correspondingly, $\frac{t_{11}}{t_{h}}=8{\left(\omega_{0}\tau \right)}^{-2}\approx 1.26\times 10^{5}\gg 1.$ This leads to a maximum allowed pressure of $P_{s}=8\left(\frac{64\nu \eta }{\pi^{2}\left(\frac{t_{11}}{t_{h}}\right)}\frac{l^{2}}{r_{0}^{4}}\right)\approx 80\;\mathrm{kPa}$, which is also amply fulfilled in all the cases studied in this work.
